# Inhibition of monocyte chemoattractant protein-1 prevents diaphragmatic inflammation and maintains contractile function during endotoxemia

**DOI:** 10.1186/cc9295

**Published:** 2010-10-07

**Authors:** Katherine Labbe, Gawiyou Danialou, Dusanka Gvozdic, Alexandre Demoule, Maziar Divangahi, John H Boyd, Basil J Petrof

**Affiliations:** 1 Meakins-Christie Laboratories, McGill University, 3626 Saint Urbain, Montreal, Quebec, Canada H2X 2P2; 2 Université Paris 6 Pierre et Marie Curie, UPRES EA2397, Service de Pneumologie et Réanimation, Groupe Hospitalier Pitié-Salpêtrière, 47-83 boulevard de l'Hôpital, 75651 Paris cedex 13, Paris, France; 3 Respiratory Division, McGill University Health Centre and Research Institute, 687 Pine Avenue West, Montreal, Quebec, Canada H3A 1A1

## Abstract

**Introduction:**

Respiratory muscle weakness is common in sepsis patients. Proinflammatory mediators produced during sepsis have been implicated in diaphragmatic contractile dysfunction, but the role of chemokines has not been explored. This study addressed the role of monocyte chemoattractant protein-1 (MCP-1, also known as CCL2), in the pathogenesis of diaphragmatic inflammation and weakness during endotoxemia.

**Methods:**

Mice were treated as follows (*n *= 6 per group): (a) saline, (b) endotoxin (25 μg/g IP), (c) endotoxin + anti-MCP-1 antibody, and (d) endotoxin + isotype control antibody. Muscles were also exposed to recombinant MCP-1 *in vivo *and *in vitro*. Measurements were made of diaphragmatic force generation, leukocyte infiltration, and proinflammatory mediator (MCP-1, IL-1α, IL-1β, IL-6, NF-κB) expression/activity.

**Results:**

*In vivo*, endotoxin-treated mice showed a large decrease in diaphragmatic force, together with upregulation of MCP-1 and other cytokines, but without an increase in intramuscular leukocytes. Antibody neutralization of MCP-1 prevented the endotoxin-induced force loss and reduced expression of MCP-1, IL-1α, IL-1β, and IL-6 in the diaphragm. MCP-1 treatment of nonseptic muscles also led to contractile weakness, and MCP-1 stimulated its own transcription independent of NF-κB activation *in vitro*.

**Conclusions:**

These results suggest that MCP-1 plays an important role in the pathogenesis of diaphragmatic weakness during sepsis by both direct and indirect mechanisms. We speculate that its immunomodulatory properties and ability to modify skeletal muscle function make MCP-1 a potential therapeutic target in critically ill patients with sepsis and associated respiratory muscle weakness.

## Introduction

Sepsis is a major risk factor for the development of critical illness myopathy [1], and impaired skeletal muscle function has been directly linked to systemic infections in humans [2]. The diaphragm is the primary muscle of respiration, and acute respiratory failure occurs in a large proportion of patients with severe sepsis [3]. Major losses of diaphragmatic force-generating capacity have been documented in several different sepsis models [4-7]. Substantial data link this decreased diaphragmatic function to the associated systemic inflammatory response syndrome (SIRS) and to the local expression of proinflammatory mediators (for example, reactive oxygen species, nitric oxide, cytokines) within skeletal muscle fibers (see reference [8] for recent review). Interestingly, evidence also indicates that the diaphragm is particularly prone to exaggerated proinflammatory gene upregulation and impaired force production during different forms of enhanced systemic inflammation [7,9,10].

Monocyte chemoattractant protein (MCP)-1, also known as CCL2, is a prototypical member of the CC subfamily of chemokines [11]. High serum levels of MCP-1 have been demonstrated in animal models of sepsis or SIRS [12-15], as well as in sepsis patients [16]. In a recent study profiling a large number of cytokines in the plasma of patients with severe sepsis, MCP-1 levels showed the best correlation with organ dysfunction and mortality [17]. MCP-1 is primarily a chemoattractant for monocytes, memory T lymphocytes, and natural killer cells, with some recent studies also pointing to a potential role in attracting neutrophils [11,18]. However, it is important to recognize that the actions of MCP-1 extend well beyond leukocyte chemoattraction. In particular, MCP-1 has important effects on the balance between pro- and anti-inflammatory cytokines [13,19,20]. In addition, MCP-1 exposure can lead to increased insulin resistance in skeletal myocytes [21] and also affects muscle repair mechanisms [22,23], suggesting a potential to significantly modify muscle function in critically ill patients.

In the present study, our principal objective was to determine whether MCP-1 is involved in the pathogenesis of diaphragmatic dysfunction associated with SIRS induced by endotoxin administration. Our principal aims were as follows: (a) to evaluate whether endotoxin administration leads to increased MCP-1 expression in skeletal muscles; (b) to assess whether an increased exposure to MCP-1 has direct effects on skeletal muscle function; and (c) to determine whether MCP-1 neutralization is able to modulate proinflammatory mediator expression and contractile function in the diaphragm during acute endotoxemic sepsis.

## Materials and methods

### Animal experiments

Experiments were performed in 8- to 10-week-old male C57BL/6 mice (Charles River Laboratories, Saint-Constant, QC, Canada). All procedures were approved by the institutional animal care and ethics committee, in accordance with the guidelines issued by the Canadian Council on Animal Care. The mice were anesthetized with a mixture of ketamine (130 μg/g) and xylazine (20 μg/g) prior to sacrifice.

#### Sepsis model

Mice were injected intraperitoneally with either *Escherichia coli *endotoxin (LPS, serotype 055:B5) (25 μg/g) or an equivalent volume of saline. Mice were sacrificed at 12 hours (unless specifically stated otherwise) after administering LPS, and the muscles (diaphragm, extensor digitorum longus (EDL), tibialis anterior) and other tissues (lungs, liver, blood) were removed for the various biochemical, histologic, and physiological analyses described later in detail. For all MCP-1 neutralization studies, the mice were pretreated with intraperitoneal injection of an anti-MCP-1 neutralizing antibody (1 μg/g) (BD Biosciences, San Diego, CA) at 12 and 24 hours before LPS administration; this antibody and dose have previously been shown to be effective in mice with septic peritonitis [14]. Control animals received the same dose of an irrelevant isotypic control immunoglobulin, administered in the same manner.

#### Local administration of MCP-1

To test the effects of exogenous MCP-1 on skeletal muscle contractility, recombinant murine MCP-1 (100 pg in 10 μl of saline) (R&D systems, Minneapolis, MN) was directly injected into the EDL muscle of the hindlimb. The contralateral EDL was injected with an identical volume of saline at the same time to serve as a within-animal control group, thereby eliminating any potential differences related to systemic absorption of the injected MCP-1. Both EDL muscles were surgically exposed to ensure an accurately placed injection, and after wound closure with sutures, the animals emerged from anesthesia and resumed normal behavior. Mice were sacrificed at 12 hours after administering MCP-1, and both EDL muscles were removed.

### Cell culture experiments

To evaluate the direct effects of MCP-1 on cytokine expression by diaphragmatic muscle cells, primary diaphragmatic muscle cell cultures were established [9] by using single living muscle fibers to isolate myoblast precursors (satellite cells). In brief, excised diaphragm muscle strips were subjected to collagenase digestion and trituration to liberate individual fibers. The individual fibers were transferred into Matrigel-coated (Becton Dickinson, Franklin Lakes, NJ) plates. Diaphragmatic myoblasts were expanded in growth medium (20% fetal bovine serum, 10% horse serum, 1% chick embryo extract in DMEM) until attaining approximately 75% confluence. The cultures were then placed in differentiation medium (2% fetal bovine serum, 10% horse serum, 0.5% chick embryo extract in DMEM) to induce myoblast fusion into differentiated myotubes. All experiments were performed on day 5 of maintenance in differentiation medium. Diaphragmatic myotubes were washed with DMEM before stimulation with recombinant murine MCP-1 (100 ng/ml).

To determine the effects of MCP-1 on NF-κB activity in muscle cells, myoblasts were simultaneously transfected with a NF-κB-driven firefly luciferase reporter plasmid (pNF-κB; Clontech, Mountain View, CA) and a constitutively active thymidine kinase promoter-driven Renilla luciferase plasmid (pRL-TK; Promega, Madison, WI), as previously described [24]. In this system, the constitutively active Renilla luciferase serves as an internal control to adjust for any differences in transfection efficiency. For these studies, we used the C2C12 skeletal muscle cell line (ATCC, Manassas, VA) rather than primary skeletal muscle cells, as the latter are known to be resistant to standard transfection techniques [25]. C2C12 myoblasts (5 × 10^5^) were seeded onto 60-mm plates and transfected the following day at approximately 50% confluence, by using Lipofectamine 2000 (Invitrogen, Carlsbad, CA). On day 5 in differentiation medium, the cells were stimulated with murine MCP-1 (100 ng/ml) (R&D Systems, Minneapolis, MN), and the activity levels of both forms of luciferase (firefly and Renilla) were quantified by using the Dual-Luciferase Reporter Assay System (Promega). Light emission was measured in an L_max _384 luminometer (Molecular Devices, Downingtown, PA), and the results are expressed as the ratio of firefly (reflecting NF-κB activity) to Renilla luciferase activities in relative light units.

### Analyses of protein and mRNA expression

A commercial ELISA kit for murine MCP-1 (R&D Systems, Minneapolis, MN) was used to measure serum and tissue MCP-1 protein levels in duplicate, according to the manufacturer's instructions. Serum was collected by cardiac puncture, and total protein was extracted from the diaphragm, tibialis anterior, liver, and lung. Frozen tissue samples were homogenized in lysis buffer (1% Triton X-100, 50 m*M *HEPES (pH 8.0), 150 m*M *NaCl, 10% glycerol, 2 m*M *EDTA, 1.5 m*M *MgCl_2_, 10 μg/ml aprotinin, 10 μg/ml leupeptin, 1 m*M *phenylmethylsulphonyl fluoride, 1 m*M *sodium orthovanadate). Homogenates were centrifuged 10 minutes at 10,000 rpm, and the supernatant protein content measured with Bradford assay (BioRad Laboratories, Hercules, CA).

To measure mRNA expression levels of MCP-1 and its receptor CCR2, IL-1α, IL-1β, and IL-6, total RNA from tissue or cell cultures was extracted by using Trizol reagent (Invitrogen) according to the manufacturer's protocol. ^32^P-labeled, anti-sense RNA probes were synthesized from commercially available Multi-Probe-Template sets (BD Biosciences, San Diego, CA). Riboprobes were hybridized overnight at 56°C with 10 μg of sample RNA, according to the manufacturer's instructions. Protected RNA fragments were separated by using a 5% polyacrylamide gel and analyzed with autoradiography. For each RNA probe, all experimental groups were run on a single gel to allow quantitative comparisons. The bands representing mRNA content were quantified by using an image-analysis system (FluorChem 8000; Alpha Innotech, San Leandro, CA), and the signals normalized to the L32 housekeeping gene as a loading control.

### Analyses of leukocyte infiltration

To quantify macrophages and neutrophils, skeletal muscle cryosections (5 μm thick) were reacted with monoclonal antibodies directed against either macrophage F4/80 (1:75 dilution) (Abcam, Cambridge, MA) or neutrophil Ly-6G (1:50 dilution) (BD Biosciences). Nonspecific binding sites were blocked by incubating sections for 1 hour with PBS containing 3% BSA and 5% goat serum, followed by goat anti-mouse IgG Fab fragment (1:20 dilution) (Jackson Laboratories, West Grove, PA) for 30 minutes. Biotinylated rabbit anti-rat IgG secondary antibody (1:100 dilution) (Vector Laboratories, Burlingame, CA) was added and revealed by using the Vectastain streptavidin-HRP system (Vector Laboratories) with DAB substrate (Sigma-Aldrich Canada, Oakville, ON, Canada). To quantify inflammatory cell infiltration, the central and adjacent 20 × fields of the tissue were photographed by using a digital camera, and a stereology software package (Image-Pro Plus; Media Cybernetics, Silver Spring, MD) was used to overlay a 275-point grid onto each image (six photographs per muscle). Inflammatory cells were quantified by using a standard point-counting method, in which an abnormal point was defined as falling either on an inflammatory cell or on a myofiber invaded by such cells. The percentage area of inflammation was then calculated by dividing the number of abnormal points by the total number of points falling on the muscle tissue section [26]. The muscle images were selected in random order, with the operator blinded to the identity of the experimental groups.

As an additional index of neutrophil activity within tissues, myeloperoxidase (MPO) activity was determined [27]. In brief, frozen tissues were homogenized in 1 ml ice-cold 50 m*M *potassium phosphate buffer at pH 6.0. Homogenates were centrifuged at 12,000 *g *for 15 minutes at 4 degrees Celsius, and the supernatant was discarded. Pellets were resuspended, homogenized, centrifuged, and the pellets were resuspended in buffer. Assays were performed in duplicate on supernatant added to buffer containing 0.167 mg/ml *o*-dianisidine and 0.0005% H_2_O_2_. Enzymatic activity was determined spectrophotometrically by measuring the change in absorbance at 460 nm over a 3-minute period. Values are expressed as units per gram of tissue, with each unit representing the change in optical density per minute.

### Muscle contractile function

The diaphragm or EDL muscle was surgically excised for *in vitro *contractility measurements, as previously described [7,28]. Muscles from the different experimental groups were selected in random order, with the individual performing the contractility measurements being blinded to their identity. After removal from the animal, muscles were transferred into Krebs solution (118 m*M *NaCl, 4.7 m*M *KCl, 2.5 m*M *CaCl_2_, 1.2 m*M *MgSO_4_, 1 m*M *KH_2_PO_4_, 25 m*M *NaHCO_3_, and 11 m*M *glucose) chilled to 4°Celsius and perfused with 95% O_2_/5% CO_2 _(pH 7.4). The muscles were then mounted in a jacketed tissue-bath chamber filled with continuously perfused Krebs solution warmed to 25°Celsius. After a 15-minute thermoequilibration period, muscle length was gradually adjusted to optimal length (L_o_, the length at which maximal twitch force is obtained). The force-frequency relation was determined by sequential supramaximal stimulation for 1 second at 5, 10, 20, 30, 50, 100, 120, and 150 Hz, with 2 minutes between each stimulation train. At the end of the experiment, L_o _was directly measured with a microcaliper and the muscle blotted dry and weighed. Specific force (force/cross-sectional area) was calculated, assuming a muscle density of 1.056 g/cc and expressed in N/cm^2^.

### Statistical analysis

All data are presented as mean values ± SD (*n *= 6 per group). Group mean differences were determined with Student's *t *test, or with one-way or two-way ANOVA with *post hoc *application of the Tukey test to adjust for multiple comparisons. A statistics software package was used for all analyses (SigmaStat V2.0; Jandel Scientific, San Rafael, CA). Statistical significance was defined as *P *< 0.05.

## Results

### Effects of sepsis on MCP-1 expression and inflammatory cells in the diaphragm

**Figure 1:**
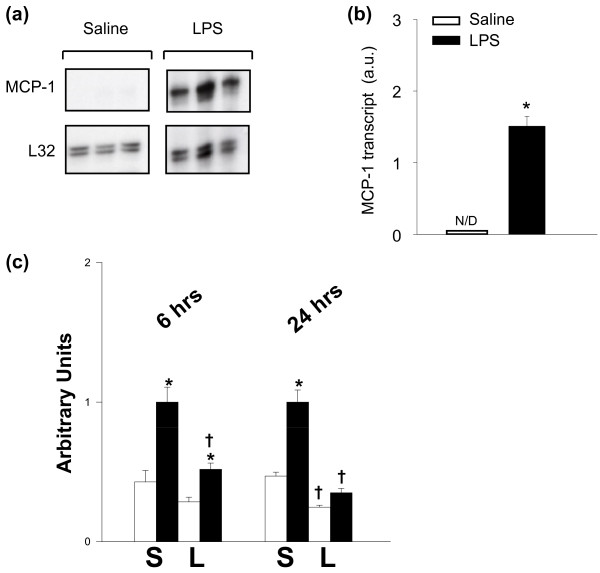
**Transcript levels of MCP-1 and its receptor in the septic diaphragm**. **(a) **Representative RNase protection assay showing MCP-1 mRNA in the diaphragm. **(b) **Quantification of MCP-1 mRNA levels in the diaphragm, normalized to the L32 housekeeeping gene. *P < 0.05 for saline versus LPS groups; N/D, not detectable. **(c) **Quantification of mRNA levels of the MCP-1 receptor, CCR2 (open bars, tibialis anterior muscle; solid bars, diaphragm; S, saline control group; L, LPS group). *P < 0.05 for tibialis versus diaphragm under the same conditions; +*P *< 0.05 for saline versus LPS groups in the same muscle.

**Figure 2:**
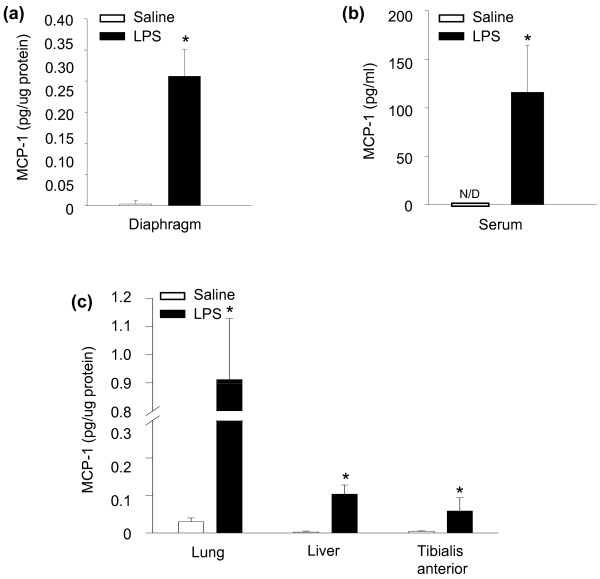
**MCP-1 protein in the diaphragm and other organs during sepsis**. MCP-1 protein content determined with ELISA in **(a) **diaphragm, **(b) **serum, and **(c) **organs and hindlimb muscle (tibialis anterior). *P < 0.05 for saline versus LPS groups. N/D, not detectable.

To evaluate mRNA expression levels of MCP-1, diaphragms from saline and LPS groups of mice were analyzed with RNase protection assay, as shown in Figure 1a. MCP-1 mRNA was not detected in control diaphragms, but was greatly increased in the diaphragms of septic animals (Figure 1b). Conversely, expression levels of CCR2, the only known receptor for MCP-1, were downregulated in the diaphragm after LPS administration (Figure 1c). The upregulation of MCP-1 mRNA transcript levels was associated with a similar increase in MCP-1 protein content within the septic diaphragm, as shown in Figure 2a. MCP-1 protein levels were also found to be significantly elevated in the serum (Figure 2b), as well as in the lung, liver, and the tibialis anterior muscle (Figure 2c) of LPS-group animals. Interestingly, MCP-1 protein levels were two- to threefold higher in the diaphragm than in the hindlimb muscle (tibialis anterior) under septic conditions.

**Figure 3:**
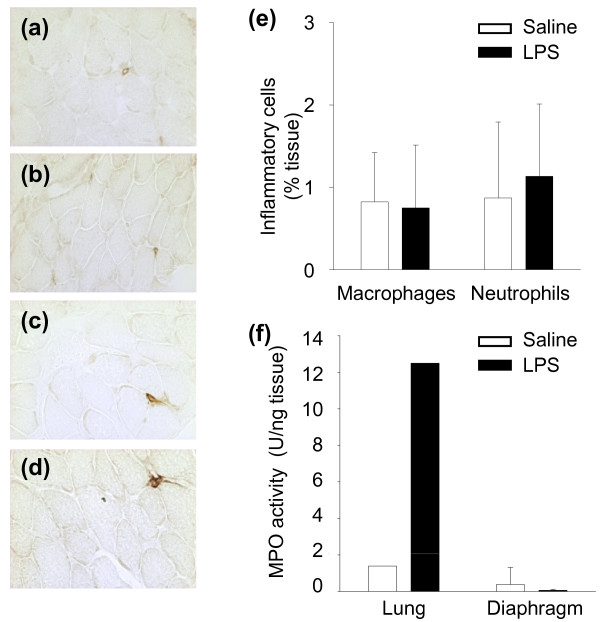
**Evaluation of inflammatory cells in the septic diaphragm**. **(a, b) **Representative F4/80 staining of macrophages in saline- and LPS-administered mice, respectively; **(c, d) **representative Ly6G staining of neutrophils in saline and LPS groups, respectively. **(e) **Morphometric quantification of macrophages and neutrophils in the diaphragm. **(f) **Myeloperoxidase (MPO) activity in the diaphragm after LPS administration.

To determine whether the augmented levels of MCP-1 detected in the septic diaphragm were associated with increased leukocyte infiltration into the muscle, immunohistochemical analysis was performed with antibodies directed against markers for macrophages and neutrophils. As shown in Figure 3, no measurable differences between control and septic diaphragms were found in the numbers of either leukocyte population. This was further confirmed for the neutrophil population by the lack of change in diaphragmatic MPO activity, whereas MPO activity was greatly increased in the lungs of septic animals (Figure 3f).

### Effects of MCP-1 on skeletal muscle proinflammatory markers *in vivo *and *in vitro*

**Figure 4:**
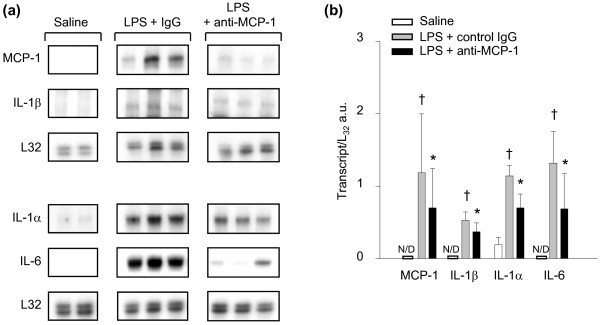
**Effects of MCP-1 inhibition on inflammatory gene expression in the septic diaphragm**. **(a) **Representative RNase protection assays showing proinflammatory gene expression in diaphragms of mice pretreated with anti-MCP-1 antibody or isotypic control antibody (IgG) during sepsis. **(b) **Quantification of proinflammatory gene mRNA levels in the diaphragm, normalized to the L32 housekeeeping gene. *P < 0.05 for IgG control antibody versus anti-MCP-1 antibody pretreatment groups; +*P *< 0.05 for IgG control antibody versus saline groups.

The ability of MCP-1 to modulate proinflammatory cytokine gene expression in the diaphragm during sepsis *in vivo *was investigated by pretreating animals with anti-MCP-1 neutralizing antibody. As indicated in Figure 4, transcript levels for IL-1α, IL-1β, and IL-6, as well as for MCP-1 itself, were all significantly lower in the diaphragms of mice that were pretreated with the MCP-1 neutralizing antibody before LPS administration. Therefore, systemic blockade of endogenous MCP-1 *in vivo *had major effects on the regulation of these proinflammatory genes in the septic diaphragm.

**Figure 5:**
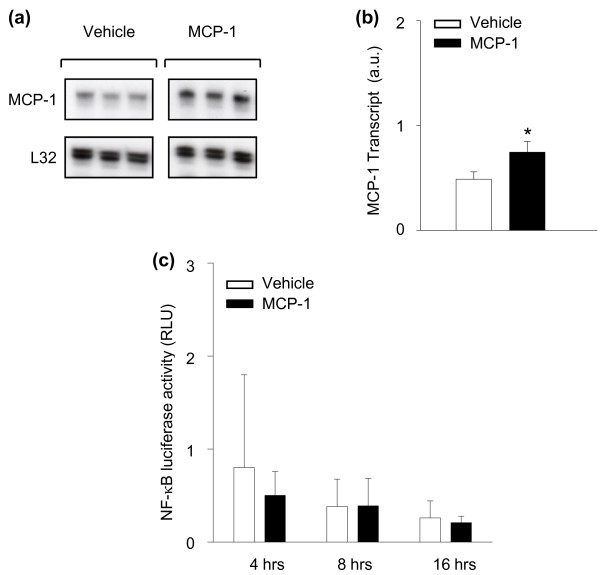
**Effects of MCP-1 treatment on inflammatory markers in cultured skeletal muscle cells**. **(a) **Representative RNase protection assays. **(b) **Quantification of MCP-1 mRNA levels, after *in vitro *stimulation of primary diaphragmatic myotube cultures with recombinant MCP-1 (100 ng/ml). **(c) **NF-κB transcriptional activity in C2C12 myotube cultures treated with recombinant MCP-1 (100 ng/ml), as determined by the plasmid transfection luciferase reporter system. *P < 0.05 for vehicle-versus MCP-1-treated groups.

We next sought to determine whether MCP-1 is capable of directly stimulating inflammatory responses in primary diaphragmatic muscle cell cultures examined at 4, 8, and 16 hours after stimulation. Interestingly, despite significant effects of MCP-1 neutralization on the expression of these genes in the septic diaphragm *in vivo*, the transcript levels of IL-1α, IL-1β, and IL-6 were unaltered by direct MCP-1 stimulation of skeletal muscle cells *in vitro *(no detectable expression under either unstimulated or stimulated conditions). As shown in Figures 5a and 5b, only MCP-1 itself was significantly upregulated by MCP-1 stimulation in diaphragmatic muscle cells, and this effect was noted at 8 hours after stimulation. Moreover, in keeping with the fact that MCP-1 did not upregulate these classic proinflammatory genes in primary muscle cell cultures, we also did not find any significant influence of MCP-1 treatment on the NF-κB transcriptional activity assay in C2C12 skeletal muscle cells (Figure 5c). Taken together, these results suggest that MCP-1 is capable of acting on skeletal muscle cells to upregulate its own expression, but in a manner not dependent on NF-κB pathway activation.

### Effects of MCP-1 on skeletal muscle contractile function *in vivo*

**Figure 6:**
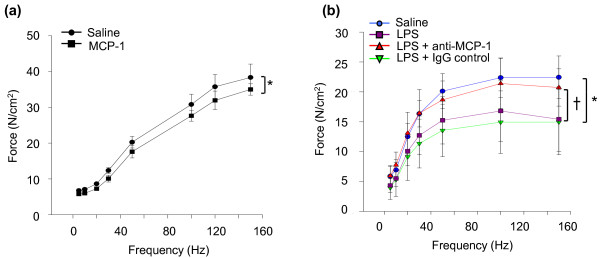
**Effects of MCP-1 modulation on skeletal muscle force-generating capacity *in vivo***. **(a) **Effects of exogenous MCP-1 injection on the force-frequency relation of the extensor digitorum longus (EDL) muscle in nonseptic mice; *P < 0.05 for saline-versus MCP-1-injected mice. **(b) **Effects of inhibiting endogenous MCP-1 on the force-frequency relation of the diaphragm in septic mice. *P < 0.05 for saline versus LPS groups; +*P *< 0.05 for IgG control antibody versus anti-MCP-1 antibody pretreatment in LPS groups.

To evaluate the potential contribution of MCP-1 to the adverse effects of sepsis on the contractile function of skeletal muscles, two different approaches were used. First, to determine whether direct exposure of skeletal muscle fibers to MCP-1 has effects on contractile function, recombinant MCP-1 protein was injected into the EDL muscle. The dose of MCP-1 administered to the EDL was extrapolated from the diaphragmatic MCP-1 content (picograms per muscle weight) at 12 hours after LPS administration, as determined with ELISA and presented earlier in Figure 2. Figure 6a shows that at 12 hours after injection of recombinant MCP-1 into the EDL, a small but statistically significant reduction was noted in the force-generating capacity of the MCP-1-injected EDL muscles relative to the contralateral control (saline-injected) muscles from the same animals. Furthermore, as was the case for septic diaphragms at the same time point after LPS administration (12 hours), the MCP-1-injected EDL muscles did not show any histologic evidence of inflammatory cell infiltration (not detected in either saline- or MCP-1-injected muscles).

Second, to determine whether MCP-1 plays a role in diaphragmatic contractile dysfunction during sepsis, the force-generating capacity of the diaphragm was compared in animals pretreated with anti-MCP-1 neutralizing antibody versus an irrelevant isotype control immunoglobulin. As expected, LPS administration led to a major decrease in diaphragmatic force production 12 hours later. The LPS-induced depression of diaphragmatic force was unaffected by pretreatment with an irrelevant isotype control antibody. In marked contrast, the loss of diaphragmatic force production at 12 hours after LPS administration was greatly alleviated in animals pretreated with anti-MCP-1 neutralizing antibody, as illustrated in Figure 6b. These findings indicate that MCP-1 plays a significant role in the impairment of diaphragmatic function associated with acute endotoxemic sepsis.

## Discussion

To our knowledge, this is the first study to examine specifically the role of a chemokine, MCP-1, in proinflammatory mediator production by the diaphragm and the contractile dysfunction of the muscle that occurs during sepsis. From a clinical standpoint, our most important observation was that neutralization of MCP-1 greatly alleviated diaphragmatic weakness in the setting of acute endotoxemia. This was associated with significantly diminished diaphragmatic expression of proinflammatory cytokines. Previous investigations in animals have shown that MCP-1 effects in sepsis can vary according to cell type and experimental model, as well as the specific mode and timing of MCP-1 inhibition. For example, in the cecal ligation/perforation (CLP) sepsis model, mice genetically deficient in MCP-1 showed lower IL-10 production in peritoneal macrophages and increased mortality [20]. In contrast, antibody neutralization of MCP-1 in the CLP context had a beneficial effect on survival [14], and the administration of an MCP-1-synthesis inhibitor, bindarit, was also reported to be beneficial in different murine models of sepsis [29]. A complex pattern of both pro- and anti-inflammatory effects on different organs has been reported after MCP-1 neutralization in CLP animals [13].

Intriguingly, in a recent prospective cohort study of patients with severe sepsis in which a multiplex analysis of 17 candidate cytokines in the serum was performed, only MCP-1 was found to be independently associated with increased mortality [17]. The fact that the diaphragm constitutively expresses CCR2 [30] led us to test the hypothesis that MCP-1 could directly regulate the expression of proinflammatory mediators in skeletal muscle cells. In keeping with this, we found that direct stimulation of primary diaphragmatic cell cultures by purified MCP-1 led to an increase of MCP-1 transcripts, suggesting the existence of positive-feedback autoregulation. Such a feed-forward loop has been previously described for other chemokines in different cell types [31,32]. Although very little is known about the mechanisms or functional significance of this positive-feedback loop, the result is likely to be an enhancement of MCP-1 actions. The downregulation of CCR2 expression that we observed in the septic diaphragm, which is analogous to that reported in monocytes exposed to LPS [33], is presumably an important mechanism for counterbalancing this effect.

Interestingly, the transcript levels of IL-1α, IL-1β, and IL-6 were unaltered by direct MCP-1 stimulation of skeletal muscle cells *in vitro*. Consistent with these findings, NF-κB reporter gene activity was also not increased in myotubes exposed to MCP-1. Although it could be argued that activation of NF-κB may have occurred more rapidly than the earliest time point examined in our study (4 hours), this appears unlikely because the firefly luciferase protein used as a readout in these experiments is stable for up to 6 hours in mammalian cells [34]. Furthermore, in primary human abdominal muscle culture, MCP-1 did not induce NF-κB activation within 1 hour of stimulation [21]. This is in contrast to the situation within isolated cardiomyocytes, in which MCP-1 (at the same dose used in our study) has been reported to upregulate IL-1β and IL-6 expression [19]. MCP-1 has also been found to stimulate the expression of IL-6 in neutrophils [18] and leukotriene B4 in peritoneal macrophages [12]. Taken together, these findings emphasize the existence of cell- and organ-specific regulatory mechanisms for MCP-1. Furthermore, given our demonstration that MCP-1 stimulation of skeletal muscle cells *in vitro *fails directly to upregulate IL-1α, IL-1β, or IL-6 expression, it is likely that the ability of MCP-1 neutralization to downregulate these cytokines *in vivo *during sepsis is achieved, at least in part, via intermediary partners.

Although MCP-1 was recently shown to play several key roles in skeletal muscle repair and metabolism [21-23,35], its influence on muscle function during sepsis has not been previously explored. We found that *in vivo *neutralization of endogenous MCP-1 during acute sepsis led to substantial decreases in the transcript levels for IL-1α, IL-1β, and IL-6, as well as MCP-1 itself, in the diaphragm. IL-1 significantly decreases muscle weight, protein content, and the rate of protein synthesis in skeletal muscle [36], whereas IL-6 can upregulate the cathepsin and ubiquitin pathways of muscle proteolysis [37]. Exposure of human skeletal muscle cells to MCP-1 at physiologic concentrations has been demonstrated to induce a state of increased insulin resistance, as indicated by alterations in insulin signaling with an associated impairment of glucose uptake [21]. Taken together, such metabolic derangements all have the potential to depress skeletal muscle contractile function. In addition, reactive oxidative species also play an important role in diaphragmatic dysfunction during sepsis [8], and overproduction of MCP-1 has been linked to increased oxidative stress and tissue damage in cardiac muscle after ischemia-reperfusion [38].

As an important leukocyte chemoattractant molecule, a plausible hypothesis was that MCP-1 overexpression in the diaphragm during sepsis might increase inflammatory cell infiltration into the muscle. This was not found to be the case, as the levels of both neutrophils and macrophages in the diaphragm were unaffected by LPS administration. In addition, although direct injection of MCP-1 into skeletal muscle was associated with a mild reduction in force-generating capacity, this was similarly not linked to increased inflammatory cell infiltration. However, this does not exclude the possibility that increased exposure to MCP-1 (either during sepsis in the diaphragm or through direct injection into the EDL) modified the activation state of resident macrophages within these muscles, and this hypothesis deserves further study.

## Conclusions

In summary, this study demonstrates that the increased endogenous MCP-1 production during SIRS induced by endotoxin contributes to proinflammatory mediator production by the diaphragm, along with a major decrease in diaphragmatic force-generating capacity. Our findings suggest that the systemic immunomodulatory properties of MCP-1, coupled with its ability to modify skeletal muscle cell function directly, could make MCP-1 an attractive therapeutic target in sepsis patients, especially in the setting of respiratory muscle dysfunction and ventilatory failure.

## Key messages

• MCP-1 is significantly upregulated in the diaphragm during acute endotoxemic sepsis

• Antibody neutralization of MCP-1 in this setting reduces the diaphragmatic expression levels of several proinflammatory cytokines that have been implicated in the pathogenesis of sepsis

• MCP-1 neutralization prevents the loss of diaphragmatic force-generating capacity normally observed during acute endotoxemia

## Abbreviations

CCL: CC chemokine ligand; CCR: CC chemokine receptor; CLP: cecal ligation and perforation; EDL: extensor digitorum longus; IL: interleukin; LPS: lipopolysaccharide; MCP: monocyte chemoattractant protein; MPO: myeloperoxidase; SIRS: systemic inflammatory response syndrome.

## Competing interests

The authors declare that they have no competing interests.

## Authors' contributions

KC was involved in all aspects of the study, GD performed muscle-contractility experiments, DG was involved in primary cell cultures, AD and MD were involved in RNase protection assays, JHB performed luciferase assays, and BJP was involved in all aspects of the study.
